# Congenital Syphilis Epidemiology, Prevention, and Management in the United States: A 2022 Update

**DOI:** 10.7759/cureus.33009

**Published:** 2022-12-27

**Authors:** Juliet Fang, Elizabeth Partridge, Geoanna M Bautista, Deepika Sankaran

**Affiliations:** 1 Center for Health and Environment, University of California Davis, Davis, USA; 2 Department of Pediatrics, University of California Davis, Davis, USA; 3 Department of Pediatrics, Division of Neonatology, University of California Davis, Davis, USA

**Keywords:** perinatal syphilis, vertical transmission, sexually transmitted infection, treponema pallidum, health disparities, sti, maternal syphilis, prenatal health, congenital syphilis

## Abstract

Congenital syphilis (CS) has dramatically increased in the United States (US) in the past decade despite the widespread availability of penicillin. Once considered an infection on the verge of elimination, CS has re-emerged as a familiar neonatal pathogen in US hospitals. This rise in cases has prompted the evaluation of potential causes and updates in prevention and management guidelines. Following a structured narrative approach, we reviewed CS data reports, peer-reviewed research articles, and updated management guidelines from state health departments over the past two decades. Our main search criteria centered on the treatment and prevention of CS, with a focus on prenatal health disparities. We identified geographical regions reporting disproportionate rates of CS, examined state laws regarding maternal syphilis testing, and evaluated potential reasons for the recent rise in cases. This article examines the current epidemiology, screening, and management recommendations for perinatal and CS in the US. It also reviews pathogenesis and clinical features in perinatal and pediatric populations. Finally, it highlights the likely contributing factors to increased CS rates and identifies areas for future research. Dramatically rising CS cases in certain regions and racial groups reflect gaps in the prevention, timely diagnosis, treatment, and management of perinatal syphilis and CS. Healthcare providers attending to mothers and children should recognize the re-emergence of this pathogen and be familiar with new screening and management guidelines. Increased federal funding for targeted interventions and research that address vulnerable populations is critical to curbing the re-emergence of this infection.

## Introduction and background

Congenital syphilis (CS), caused by the transmission of *Treponema pallidum* from mother to child, was considered a closely controlled infection, with less than 500 annual CS cases consistently reported at the start of the 21st century [1]. However, since 2012, we have observed dramatic increases in this infection and its associated morbidity and mortality [2,3]. From 2012 to 2020, the incidence of CS increased from 8.4 cases per 100,000 live births to 57.3 cases per 100,000 live births, a nearly seven-fold increase [2]. The rise of CS has occurred despite the widespread availability of penicillin, the definitive treatment for maternal and perinatal syphilis, and the public health infrastructure of a highly developed nation. The evolving epidemiology of CS within the United States (US) highlights domestic health disparities and has led to updated screening and treatment recommendations that will be outlined in this review. 

## Review

Microbiology and clinical presentation

**Figure 1:**
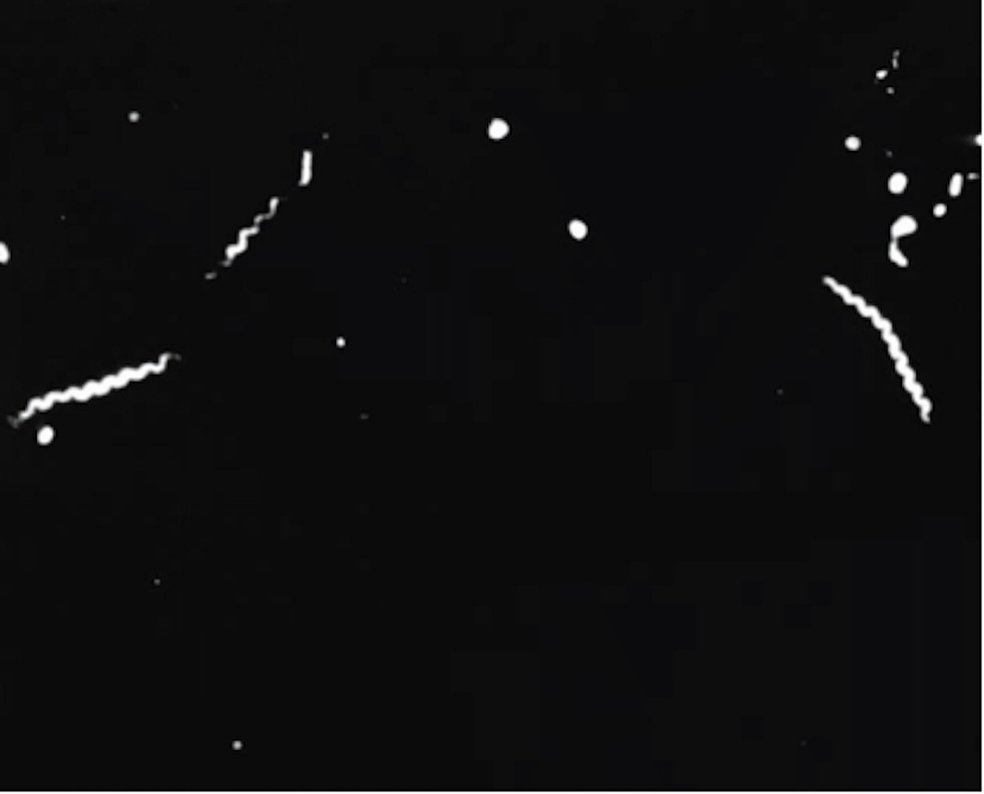
Dark field visualization of Treponema pallidum bacterial spirochetes. Public domain photo courtesy of W.F. Shwartz and the Centers for Disease Control and Prevention Public Health Image Library, 1961.

*Treponema pallidum* subspecies *pallidum* *(T. pallidum*), the bacterial spirochete that causes syphilis, was first identified by Fritz Schaudinn and Erich Hoffman in 1905 [4]. Most *Treponema* species are not easily detected by routine Gram stain. *T. pallidum* is identified with dark field microscopy of clinical samples from the syphilitic lesion exudate. *Treponema* appears helically coiled and corkscrew-shaped on dark field microscopy (Figure 1) [5].

Syphilis is a sexually transmitted infection (STI) spread through contact with a chancre, condyloma lata (painless, hypertrophic papular lesions of varying sizes), or through vertical transmission, which occurs when the pathogen is passed from mother to child before or after birth. Syphilis is classified into four stages of infection: primary, secondary, latent, and tertiary [6]. Neurosyphilis can develop at any stage of infection. Primary syphilis infection begins when *T. pallidum* penetrates dermal microabrasions or intact mucous membranes, often resulting in a chancre at the site of inoculation and enlarged adjacent lymph nodes. These lesions typically appear three weeks after exposure (range 10-90 days) and spontaneously resolve within a few weeks [6]. The chancre and regional lymphadenopathy are painless and sometimes inconspicuous to the individual, which can delay diagnosis. Secondary syphilis occurs one to three months after the initial infection is left untreated and can include a flu-like illness with headache, sore throat, generalized lymphadenopathy, myalgias, and arthralgia. Skin findings include a generalized maculopapular rash involving the palms and soles and condylomata lata found in warm, moist regions (e.g., perineum). Without treatment, symptoms of secondary infection resolve over 3-12 weeks. Following the secondary stage, untreated patients develop latent syphilis (early latent or late latent), during which their symptoms disappear. Patients are considered to have an early latent infection if transmission occurred within the preceding year. Patients infected for greater than one year (or if the duration of infection is unknown) are considered to have late latent infection. The resolution of symptoms seen in patients with latent disease can mislead infected individuals into believing they are no longer infected or infectious. However, if the patient remains untreated, the infection can progress to tissue and bone destruction (gumma), characteristic of tertiary syphilis, which may occur 15-30 years after the initial infection [6]. 

Perinatal syphilis occurs when a pregnant individual contracts syphilis. The clinical features of perinatal syphilis are similar to those in non-pregnant people (Table 1).

**Table 1:** Stages and treatment of maternal syphilis. IM: intramuscular; IV: intravenous Created with Biorender.com by G.M. Bautista

Stage	Symptom Onset	Clinical Features	Treatment
Primary	3 weeks	Painless chancre; spontaneous resolution in several weeks	Benzathine penicillin IM x 1
Secondary	1 to 4 months	Flu-like illness, generalized lymphadenopathy, myalgias, arthralgies, rash, condylomata lata; spontaneous resolution in 3 to 12 weeks	Benzathine penicillin IM x 1
Early latent	≤ 1 year	None	Benzathine penicillin IM x 1
Late latent	> 1 year	None	Benzathine penicillin IM x 3 (dosed one week apart)
Tertiary	15 to 30 years	Gumma (granulomatous growths), aortitis, dementia, tabes dorsalis	Benzathine penicillin Im x 3 (dosed one week apart)
Neurosyphilis	Any time	Meningitis, seizures, uveitis, optic atrophy, hearing loss	Aqueous penicillin IV x 10 to 14 days

A pregnant mother with syphilis can transmit the spirochete to her child in utero via placental blood flow or through direct contact with an infected lesion during childbirth. In addition, exposure to maternal syphilis at any stage may result in CS infection in the neonate [7]. The risk of maternal-to-neonatal transmission in a child born to an untreated mother is correlated with the stage of maternal infection and gestational age at the time of maternal infection. For example, primary or secondary infection carries a transmission rate of 60-100%, while approximately 40% and <8% transmission occur in mothers with early latent and late latent infection, respectively [3]. In other words, babies born to mothers who acquire syphilis during the third trimester carry the greatest risk of developing congenital infection [3,8]. Penicillin is the only antibiotic that can treat perinatal syphilis and cross the placenta at sufficient levels to prevent or treat infection in the fetus. At this time, there are no proven alternatives to penicillin [3]. Early identification and treatment of early-stage perinatal syphilis are 98% effective in preventing the mortality and morbidity associated with congenital infection [9]. 

**Figure 2:**
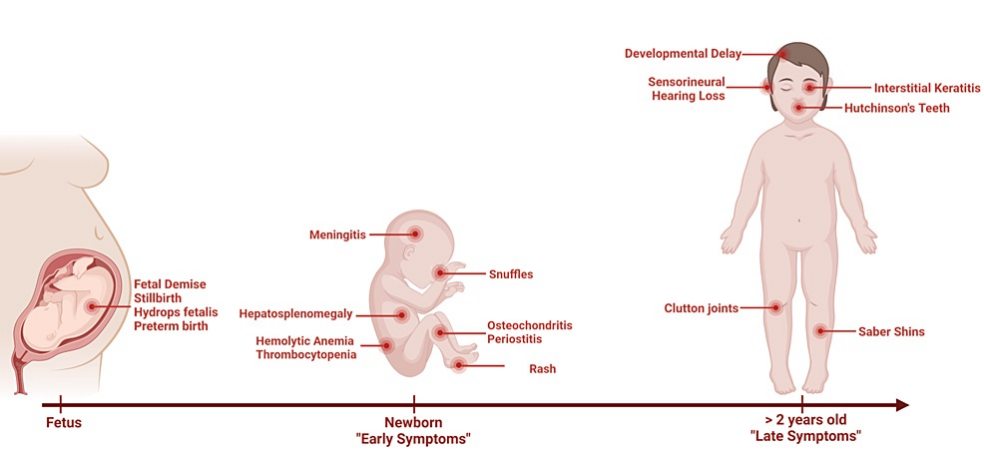
Schematic of congenital syphilis infection. Created with Biorender.com by G.M. Bautista.

CS is classified as an early or late disease (Figure 2). Early disease (ranging from birth to two years) commonly manifests within the first three months of life. Clinical manifestations of early infection include stillbirth (up to 40% of untreated pregnancies), hydrops fetalis, preterm birth, central nervous system infection (manifesting as meningitis, uveitis, optic atrophy, seizures, and hearing loss), hepatosplenomegaly, hyperbilirubinemia, cholestasis, hemolytic anemia, thrombocytopenia, snuffles (nasal congestion and excessive nasal discharge), generalized lymphadenopathy, pneumonia, osteochondritis or periostitis, pseudoparalysis of Parrot (inability to move an extremity due to painful periostitis), mucocutaneous lesions or maculopapular rash, and desquamation involving the palms and soles [3]. 

The majority, up to 70%, of infected neonates are asymptomatic, placing them at risk of missed or delayed diagnosis and inadequate treatment [10]. If untreated, asymptomatic infected infants can develop late (> two years) clinical manifestations, including the classic triad of CS: interstitial keratitis, sensorineural hearing loss, and notched central incisors (Hutchinson teeth). Other late signs of CS include developmental delay, anterior bowing (saber) shins, painless knee swelling (Clutton joints), frontal bossing, mulberry molars (multiple rounded rudimentary enamel cusps affecting the permanent first molars), and saddle nose (collapsed nose bridge) (Figure 2) [3]. 

Epidemiological trends

In the early 2000s, syphilis rates were very low due mainly to widespread and well-funded sexual health education, STI prevention (i.e., screening, contact tracing), and treatment programs [11]. This led to the belief that syphilis could be eradicated. However, since 2012, rates of syphilis and CS have dramatically increased, with the Centers for Disease Control and Prevention (CDC) reporting a record-breaking number of CS cases nationally (2148) in 2020 [12]. This included 149 CS-related stillbirths and infant deaths in the US. Preliminary data for 2021 estimate 2677 CS cases nationally, a sevenfold increase in cases since 2012 (332) [2]. 

The burden of CS in 2020 was not equal between geographical regions or racial groups. Almost half (48.5%) of all CS cases in 2020 were reported from Texas and California [13]. New Mexico (182.9 cases per 100,000 live births), Arizona (151.2 cases per 100,000 live births), and Texas (148.6 cases per 100,000 live births) reported the highest CS incidence rates in the same year. Black (746) and Hispanic/Latino (637) populations shared a disproportionate burden of CS cases in 2020. The CS incidence rate for Black individuals increased almost threefold between 2016 and 2020 (46.7 to 134.9), while that of Hispanic/Latino individuals increased approximately by 3.5 times (20.6 to 71.9). These increases reflect the historical trend that populations of color have a higher rate of STI infection than White populations. Structural and interpersonal racism drives this disparity, as social inequities experienced among Black and Hispanic/Latino populations give rise to risk factors for high-risk sexual behavior, such as substance abuse, domestic violence, unstable housing, and other STIs, such as human immunodeficiency that increase the likelihood of CS [14]. It was racist stereotypes about the sexuality of the Black population that led to the inhumane Tuskegee Experiment, in which Black males were deceived and denied treatment for syphilis, only one of many instances that have increased distrust of the medical community amongst Black populations. Today, fear of negative stereotypes may dissuade individuals from seeking out prenatal treatment, leading to an increased risk of CS. 

The recent increase in syphilis and CS infections is multifactorial. In addition to ongoing social and health inequities such as poverty (contributing to reduced healthcare access and transportation) and racism in medical care, stagnant federal funding for STI prevention likely contributes to an increase in CS cases. Federal funding for STI prevention has experienced a 40% reduction in the past two decades while STI cases have soared [15]. The vast majority of clinics and testing centers have subsequently reported closing or severely limiting hours or staff, thereby decreasing opportunities for testing and treatment [15]. Reduced funding may also result in a lack of patient follow-up and contact tracing, which are essential strategies for CS prevention. According to a 2018 Morbidity and Mortality Weekly Report, the most common missed opportunity for CS prevention was a lack of adequate maternal syphilis treatment despite receipt of a timely syphilis diagnosis in 31% of mothers with perinatal syphilis [14]. This was followed by a lack of timely prenatal care in 28% and late identification of seroconversions in 11% [14]. Entry gaps to prenatal healthcare, retention gaps between prenatal visits, syphilis testing, and treatment appointments are obstacles to care, especially in at-risk populations struggling with poverty or substance use [16]. Persons who use substances of abuse during pregnancy may also avoid prenatal healthcare due to fear of judgment and legal consequences from healthcare providers, further increasing the risk of delayed diagnosis and missed or delayed treatment for this particularly vulnerable group [16]. Methamphetamine and heroin use have been identified as risk factors [1] for acquiring syphilis during pregnancy and may partially explain the high concentration of CS cases in the Southwestern US [17]. 

Public health interventions

In response to the alarming rise in syphilis and other STIs, the US Department for Health and Health Services launched the Sexually Transmitted Infections National Strategic Plan in January 2021 [18]. The plan aims to guide public health, government, community-based, and academic stakeholders to create, enhance, and expand STI prevention and treatment efforts over the next five years. This plan prioritizes STI prevention among pregnant individuals. In addition, the American College of Obstetrics and Gynecology, American Academy of Pediatrics (AAP), and the American Academy of Family Physicians align with the CDC and US Preventive Services Task Force recommendations.

At the local level, several state departments have developed public health interventions targeting at-risk populations. For example, the Department of State Health Services in Texas, a state with consistently high CS incidence rates, launched a podcast in February 2022 designed to spread awareness about the management and prevention of syphilis and CS [19]. Oklahoma, another state with high CS incidence, has also encouraged collaboration between the Oklahoma State Department of Health and the state’s health care providers through planning and strategizing sessions to address CS [20]. Louisiana, which passed a state law requiring syphilis screening in the first and third trimesters of pregnancy in 2014, created nine regional CS case review boards composed of healthcare providers and disease intervention specialists to review every CS case and assess for potential missed opportunities for prevention [21]. These are three examples of innovative CS-directed intervention strategies states have implemented in the past decade. Additional federal funding for STI prevention and treatment efforts is critical to ensure improved patient follow-up and staff support for perinatal and CS. Funding is also needed for future research to evaluate the socioeconomic [22] and political factors contributing to the disproportionate CS case burden in the Southwest region of the US. For example, identifying specific subpopulations, such as persons using substances of abuse and migrant workers who lack citizenship in specific areas that experience high rates of CS, may also guide future health policy [23]. 

Perinatal syphilis prevention and management 

Per the CDC, syphilis screening should use nontreponemal tests such as rapid plasma regain (RPR) at three different time points during pregnancy (the first prenatal visit, third trimester, and at delivery) for pregnant people residing in areas with high endemic rates of syphilis or for persons with the following risk factors for syphilis infection: multiple sexual partners, drug use, or housing insecurity [1] Geographical areas with a high prevalence of syphilis have been identified in the US. As of December 2022, syphilis screening at the initial prenatal visit is mandatory in 84% of states, including the five states with the highest incidence rates of CS (New Mexico, Arizona, Texas, Nevada, and Oklahoma). Eight states (Hawaii, Iowa, Maine, Minnesota, Mississippi, New Hampshire, North Dakota, and Wisconsin) do not legally require syphilis screening at the initial prenatal visit. Twenty-eight percent of states require syphilis screening during the third trimester, and only five states (Alabama, Arizona, New Jersey, North Carolina, and Texas) require screening at the time of delivery. New Mexico, with the highest CS incidence rate in 2020 (182.9 per 100,000 live births), requires screening only at the first prenatal appointment [1]. 

For pregnant individuals with risk factors for syphilis infection (multiple sex partners, drug use, housing insecurity), holistic, nonjudgmental, and enhanced outpatient preventative strategies are encouraged. This approach includes prenatal screening for syphilis at the first point of medical contact (i.e., emergency rooms, pregnancy testing sites, primary care clinics, vaccination clinics, mental health clinics, and detention facilities), targeted education for front-line healthcare providers on diagnosis and follow-up of pregnant patients at risk of syphilis infection, and consistent monitoring of public and private laboratories to ensure the prompt and thorough reporting of perinatal syphilis. In addition, mobile clinics for substance use and HIV testing may serve as high-impact locations for screening at-risk populations that may otherwise not seek timely prenatal care [24]. 

For the diagnosis of perinatal syphilis (not for CS), a combination of treponemal and non-treponemal tests are used. Treponemal tests (e.g.: *T. pallidum* particle agglutination (TP-PA) test, fluorescent treponemal antibody absorption test (FTA-ABS), *T. pallidum* enzyme immunoassay (TP-EIA), *T. pallidum* chemiluminescence assay (TP-CIA)) detect antibodies against *T. pallidum*. Whereas non-treponemal tests (e.g.: VDRL, RPR) identify antibodies to biomarkers released during cellular damage as a result of *T. pallidum* infection. Treponemal tests will remain positive for life despite successful treatment, except for a minority (15-25%) of patients treated during primary infection who revert to non-reactive treponemal testing after two to three years. A negative treponemal test generally indicates no infection. Thus, in a pregnant woman with no risk factors for syphilis and a negative treponemal test, a positive non-treponemal test is likely a false positive. False positive nontreponemal (RPR) test results can occur in the setting of certain infections (Epstein-Barr virus, hepatitis, HIV, varicella, measles, tuberculosis, malaria, endocarditis) in patients with lymphoma, connective tissue disorders, and advanced age. Pregnancy and injection drug use are also risk factors for false positive RPR results. False negative nontreponemal (RPR) test results can be seen in early/incubating primary syphilis, untreated acquired or CS of long duration, or the prozone phenomenon (serum containing high concentrations of antibody against *T pallidum*). Non-treponemal tests are used in isolation to diagnose CS and to track response to treatment in the mother and infant. In adult patients with adequately treated perinatal infection, RPRs should decrease by fourfold within 12-24 months and eventually become nonreactive [3]. However, some adequately treated patients maintain low non-treponemal (RPR or VDRL) test titers for several years, a state referred to as serofast [3]. In infants appropriately treated for CS, RPRs should be nonreactive within 12 months of life. In 2021, the AAP guidelines specified that a repeat lumbar puncture is unnecessary for infants with abnormal CSF indices at diagnosis as long as the infant received ten days of aqueous penicillin and repeat serum non-treponemal testing (RPR) is nonreactive by age 12 months. 

Treatment regimens range from a single dose to three weekly doses depending on the stage of syphilis infection. Specifically, early-stage syphilis (primary, secondary, and early latent) requires only a single dose of 2.4 million units of benzathine penicillin G, while late latent, unknown duration, or tertiary syphilis is treated with three doses of penicillin G administered weekly for three weeks [1]. The treatment for neurosyphilis is 10-14 days of IV aqueous penicillin, as benzathine penicillin does not achieve optimal levels in the central nervous system. There are no proven alternatives to penicillin G for treating syphilis during pregnancy. For mothers allergic to penicillin, desensitization is recommended, followed by definitive treatment with penicillin G [25]. For those receiving treatment, serial non-treponemal testing (i.e., RPR) is recommended for monitoring treatment response and timely diagnosis of reinfection. Timely identification and treatment of partners are critical to preventing reinfection in pregnant persons after initial treatment of perinatal syphilis [14].

Screening and treatment monitoring can be particularly challenging for pregnant people with risk factors for syphilis infection. For example, focus groups with pregnant women and semi-structured interviews with prenatal care providers on the gaps in CS prevention in Kern County, California, found that underserved pregnant people experienced disproportionate challenges in access to care, and social, economic, and cultural barriers significantly impacted patient retention. Thus, it is imperative that prenatal care providers are trained to identify at-risk individuals and that clinics and hospitals are supported with resources to maintain patient follow-up and continued care [16].

CS prevention and management 

The CDC and AAP have published algorithms for evaluating and managing infants born to mothers with syphilis infection during pregnancy [1,3]. All infants born to mothers with reactive non-treponemal tests (i.e., RPR) at any point during pregnancy should receive syphilis testing by a non-treponemal test (RPR) at the time of birth. Babies are stratified into risk scenarios (CDC) or categories (AAP) for CS infection based on the infant’s physical exam findings, infant RPR compared to maternal RPR at the time of delivery, maternal treatment history, and maternal RPR titers. Risk stratification informs the infant workup (i.e., complete blood count, lumbar puncture for cerebrospinal fluid analysis, bone radiographs to evaluate for osteolytic lesions) and treatment options which include 10 days of aqueous IV penicillin versus a single dose of intramuscular benzathine penicillin versus no antibiotic treatment [1,3]. Table 2 summarizes the CDC scenarios and AAP risk categories and their respective evaluation and treatment recommendations.

**Table 2:** Treatment scenarios and risk categories for congenital syphilis. CSF: cerebral spinal fluid; CBC: complete blood count; NI PE: normal physical exam; tx: treatment; IM: intramuscular; RPR: rapid plasma reagin. *Abnormal physical exam (Abnormal PE) that is consistent with congenital syphilis; **CSF cell count, protein, Venereal Disease Research Laboratory test; ^π^Other tests as clinically indicated (e.g., liver function tests, neuroimaging, ophthalmologic exam, auditory brain stem response)

Scenario (CDC) Risk Category (AAP)	Finding	Evaluation	Treatment
Scenario: 1, Category: proven or highly probable congenital syphilis	Abnormal PE* OR Titer ≥4-fold maternal titer	CSF**, CBC, Long-bones, Other^π^	Penicillin G x 10 days (regardless of evaluation results)
Scenario: 2, Category: proven or highly probable congenital syphilis	NI PE AND Titer ≤4-fold maternal titer AND Maternal tx: none/ unknown/ inadequate or initiated <30 days before delivery	CSF**, CBC, Long-bones	Penicillin G x 10 days (if evaluation is abnormal, uninterpretable, incomplete, or follow-up uncertain OR Benzathine Penicillin IM x 1 (evaluation and follow-up certain)
Scenario: 3, Category: less likely congenital syphilis	NI PE AND Titer ≤4-fold maternal titer AND Maternal tx: adequate and initiated ≥ 30 days before delivery and no concern for re-infection	None	Benzathine Penicillin IM x 1 (if follow-up uncertain) OR No tx with follow-up titers
Scenario: 4, Category: unlikely congenital syphilis	NI PE AND Titer ≤4-fold maternal titer AND Maternal tx: adequate before pregnancy	None	No tx with follow-up titers (if infant RPR positive) OR Benzathine Penicillin IM x 1 (if follow-up uncertain)

Infants receiving IV penicillin therapy require hospital admission to complete treatment. Outpatient follow-up with RPR testing is mandated for all babies born to mothers with perinatal syphilis with reactive infant RPR at the time of delivery (regardless of treatment received) and for babies with nonreactive RPR tests who are at risk for incubating syphilis. The baby’s RPR should be repeated in two to three months and followed outpatient by the pediatrician or pediatric infectious diseases specialist until it becomes nonreactive [1,3].

For newborns who stratify into CDC scenario 2 (AAP category of possible CS), information on maternal syphilis infection and treatment is critical to informing the child’s evaluation and treatment. Of note, syphilis is a reportable condition. For mothers who present with unclear testing or treatment history, the mother’s county public health department(s) can be a valuable source of this information. Additionally, maternal treatment of perinatal syphilis with penicillin (single IM dose for primary/secondary/early latent syphilis and three weekly doses for latent/tertiary syphilis) is considered adequate if the mother initiates (i.e., receives one dose) treatment at least 30 days before delivery [1].

Maternal treatment failure or reinfection should be considered under the following circumstances: (i) maternal symptoms persist or recur, (ii) maternal RPR shows a sustained (at least two weeks) fourfold or greater increase, (iii) there is epidemiological data to support possible reinfection including unprotected sexual contact with an untreated/undertreated partner or a new partner of unknown syphilis status, and (iv) maternal RPR fails to decline fourfold within 12-24 months [3]. Of note, mothers with low RPR titers or those diagnosed after the first trimester may not have a fourfold decrease in titer at the time of delivery, despite adequate treatment. Additionally, some patients remain with low and stable VDRL and RPR titers before and during pregnancy and after delivery (e.g., VDRL ≤1:2; RPR ≤1:4) [13].

## Conclusions

CS has re-emerged as a public health issue that can result in high mortality and severe morbidity in affected children. The resurgence of syphilis in the US is multifactorial and has resulted in updated prevention and management strategies. This includes more frequent screening for perinatal syphilis at the first prenatal appointment, third trimester, and delivery. This also includes targeted screening and follow-up for at-risk populations at the first point of medical contact like emergency rooms, pregnancy sites, drug rehab, detention, and mental health facilities. Additionally, all infants born to mothers with reactive non-treponemal tests at any point during pregnancy should be evaluated for CS before discharge from the birth hospital and receive follow-up testing until non-treponemal tests are non-reactive. Health care providers who are knowledgeable about their local syphilis epidemiology, at-risk populations and updated management guidelines can improve maternal and neonatal outcomes.
